# Casagranda F, Luz VG, Martins CP, Dias-Scopel RP, Fernandes R, Fonseca W. A saúde indígena na atenção especializada: perspectiva dos profissionais de saúde em um hospital de referência no Mato Grosso do Sul, Brasil. Cad Saúde Pública 2024; 40(6):e00094622.

**DOI:** 10.1590/0102-311XER094622

**Published:** 2024-09-09

Onde se lê:

^2^ Instituto Leônidas & Maria Deane, Fundação Oswaldo Cruz, Manaus, Brasil.

Leia-se:

^2^ Fiocruz Mato Grosso do Sul, Fundação Oswaldo Cruz, Campo Grande, Brasil.

